# Flexible Supercapacitors Based on Polyaniline Arrays Coated Graphene Aerogel Electrodes

**DOI:** 10.1186/s11671-017-2159-9

**Published:** 2017-06-08

**Authors:** Yu Yang, Yunlong Xi, Junzhi Li, Guodong Wei, N. I. Klyui, Wei Han

**Affiliations:** 1 Key Laboratory of Physics and Technology for Advanced Batteries (Ministry of Education), Jilin University, Changchun, 130012 People’s Republic of China; 2 International Center of Future Science, Jilin University, Changchun, 130012 China; 3 Institute of Semiconductor Physics, National Academy of Sciences of Ukraine, Pr. Nauki 41, 03028 Kyiv, Ukraine

**Keywords:** Supercapacitor, Electrodeposition, rGO aerogel, Polyaniline array, Flexible

## Abstract

Flexible supercapacitors(SCs) made by reduced graphene oxide (rGO)-based aerogel usually suffer from the low energy density, short cycle life and bad flexibility. In this study, a new, synthetic strategy was developed for enhancing the electrochemical performances of rGO aerogel-based supercapacitor via electrodeposition polyaniline arrays on the prepared ultralight rGO aerogel. The novel hybrid composites with coated polyaniline (PANI) arrays growing on the rGO surface can take full advantage of the rich open-pore and excellent conductivity of the crosslinking framework structure of 3D rGO aerogel and high capacitance contribution from the PANI. The obtained hybrid composites exhibit excellent electrochemical performance with a specific capacitance of 432 F g^-1^ at the current density of 1 A g^-1^, robust cycling stability to maintain 85% after 10,000 charge/discharge cycles and high energy density of 25 W h kg^-1^. Furthermore, the flexible all-solid-state supercapacitor have superior flexibility and outstanding stability under different bending states from the straight state to the 90° status. The high-performance flexible all-solid-state SCs together with the lighting tests demonstrate it possible for applications in portable electronics.

## Background

The increasing demand for modern electronics such as display panels, light-emitting diodes (LEDs) and various sensors have facilitated the rapid advancement of flexible energy storage devices. Flexible supercapacitors (SCs) as an important member of energy storages family have attracted more and more concentration due to their sensational capacity performance, high power density and energy density compared to traditional capacitor and batteries, respectively [1–4]. By far, in spite of the obvious advances, flexible SCs utility are greatly limited because of the relatively poor performance of the electrode materials, so the choice of electrode materials is still very important [5–9].

Until now, electrode materials are mainly divided into three main groups: carbon materials, metal oxides, and conductive polymer. Among them, carbon-based materials for electrical double-layer capacitors (EDLCs) possess the advantages of the large specific surface area, high electroconductivity, and long cycle stability, however, the low specific capacitance has limited them further application [10–12]. On the contrary, the metal oxides and conductive polymer for pseudocapacitors have the high specific capacitance due to the extra capacitance contribution from faradic reaction in the charge-discharge process, but the short cycle life hinders these material-based SC developments [13]. Therefore, extensive reports have been presented to synthesis the nanocomposites of carbon materials and metal oxides/conductive polymer materials owing to their combining unique properties of individual nanostructures and possibly synergistic effects. For example, He et al. [14] fabricated 3D graphene-MnO_2_ composite networks using the method of chemical vapor deposition (CVD) and electrochemical deposition and its specific capacitance is of 465 F g^-1^ with cycle performance of 81.2% (5000 cycles). Meng et al. [15] developed 3D rGO-PANI film by template filtration and polymerization which provide a specific capacitance value up to 385 F g^-1^ at the current density 0.5 A g^-1^. Xin et al. fig prepared a graphene-based composite by the in-situ growth of a self-supporting graphene on a flexible graphite sheet via electrochemical intercalation and then the electrodeposited the polyaniline on the surface of graphene, the prepared electrode has a specific capacitance 491.3 F g^-1^. Although those nanocomposites exhibit the excellent electrochemical performance, little attention has been devoted to the mechanical property of the electrodes, which also play a crucial role, especially for flexile SCs.

In this study, novel flexible all-solid-state supercapacitors based on 3D rGO aerogel/polyaniline array hybrid electrodes were fabricated via a mechanical pressing and followed by electrodeposition process. The ultralight 3D rGO aerogel with excellent mechanical property, which could sustain a 4000 times of its original weight and stand on the stamen of flower, can be used as the ideal framework for growth of the PANI array, facilitating the enhanced mechanical stability of flexible all-solid-state electrode. The hybrid composites were further demonstrated with advantages of high specific capacitance of 432 F g^-1^, excellent rate capability (81.4% after the current density increases 20 times), and good energy density (25 W h kg^-1^ at the power density of 681 W kg^-1^). More importantly, the developed all-solid-state SCs have superior flexibility and outstanding stability under different bending states status with long time measurements.

## Methods

### Synthesis of 3D rGO Aerogel

The 3D rGO aerogel was synthesized by one-step self-assembled hydrothermal process [16]. A 60 mL of 2 mg mL^-1^ homogeneous GO aqueous dispersion was sealed in a 100 mL Teflon-lined autoclave and maintained at 180 °C for 12 h. Then the autoclave was naturally cooled to room temperature and the as-prepared rGO hydrogels were taken out with a filter paper to remove surface water. Subsequently, the as-prepared rGO hydrogels were cut into small slices with a diameter of about 10 mm and thickness of about 1 mm and undergo freeze-drying under −83 °C for 48 h. Then, with the assistance of roller press the 3D-rGO slice was pressed directly onto the stainless steel wire mesh (the size of active material was 1 × 1 cm) and the 3D-rGO-based aerogel was obtained.

### Electrodeposition Process for Growth of Flexible Hybrid Composites

The electrodeposition experiments were carried out in a three-electrode configuration with the as-prepared 3D-rGO film as the working electrode, a Pt plate as the counter electrode, and Hg/Hg_2_SO_4_ (sat. K_2_SO_4_) electrode as the reference electrode. The electrolyte was mixed with 0.05 M aniline and 1 M H_2_SO_4_ solution. The electrodeposition was performed at a current density of 2 mA · cm^-2^ for 7000 s at room temperature. The area of 3D-rGO employed for electrodeposition PANI was 1 × 1 cm. After washed with water, absolute ethyl alcohol, and dried at room temperature in vacuum oven for 24 h, the hybrid composites were prepared. For comparison, the aniline arrays prepared by electro-polymerization were directly growth on the stainless steel wire in the same way.

### Characterization

The surface morphology and microstructure of the samples were investigated by scanning electron microscopy (SEM, MAGELLIAN-400) and transmission electron microscope (TEM, JEOL JSM-2010 F), respectively. X-Ray Diffraction (XRD) was recorded on a Japan Rigaku 2550 X-ray powder diffractometer system with Cu Kα radiation (λ = 1.54056 Å) operating at 40 kV, 250 mA and the scanning angle from 10° to 70°. The Raman spectra were collected by Raman spectroscopy (Renishaw), using a 514 nm laser to identify the molecular structure of the samples. The X-ray photoelectron spectroscopy tests (XPS) were measured with a VG ESCALAB MK II electron spectrometer to characterize the surface chemical states of the samples. Electrochemical experiments of the samples were carried out by using a CHI 760E electrochemical workstation (Shanghai Chenhua Instrument Company Instruments, China) and an electrochemical workstation (IVIUM, Netherlands) at ambient temperature (about 20 °C).

### Calculation

The specific capacitances were calculated from the discharge curves according to the formula as follows:

1 $$ C=\frac{I\times \varDelta \mathrm{t}}{\mathrm{m} \times \varDelta \mathrm{V}} $$

where *C* (F g^−1^) is the specific capacitance of the sample, *I* (A) is discharge current, *Δt* (s) is the discharge time, *m* (g) is the mass of the active material, and *ΔV* is the potential drop during discharge.

The energy density and the power density based on flexible all-solid-state SCs can be calculated from the following equations:

2 $$ E=\frac{C\times \varDelta {\mathrm{V}}^2}{2} $$

3 $$ P=\frac{E}{t} $$

Where *E* is the energy density (W h kg^-1^), *P* is the power density (W kg^-1^), *C* presents the total capacitance of the flexible all-solid-state SCs, *∆V* is the potential drop during discharge process, and *t* is the discharge time [17].

## Results and Discussion

**Fig. 1:**
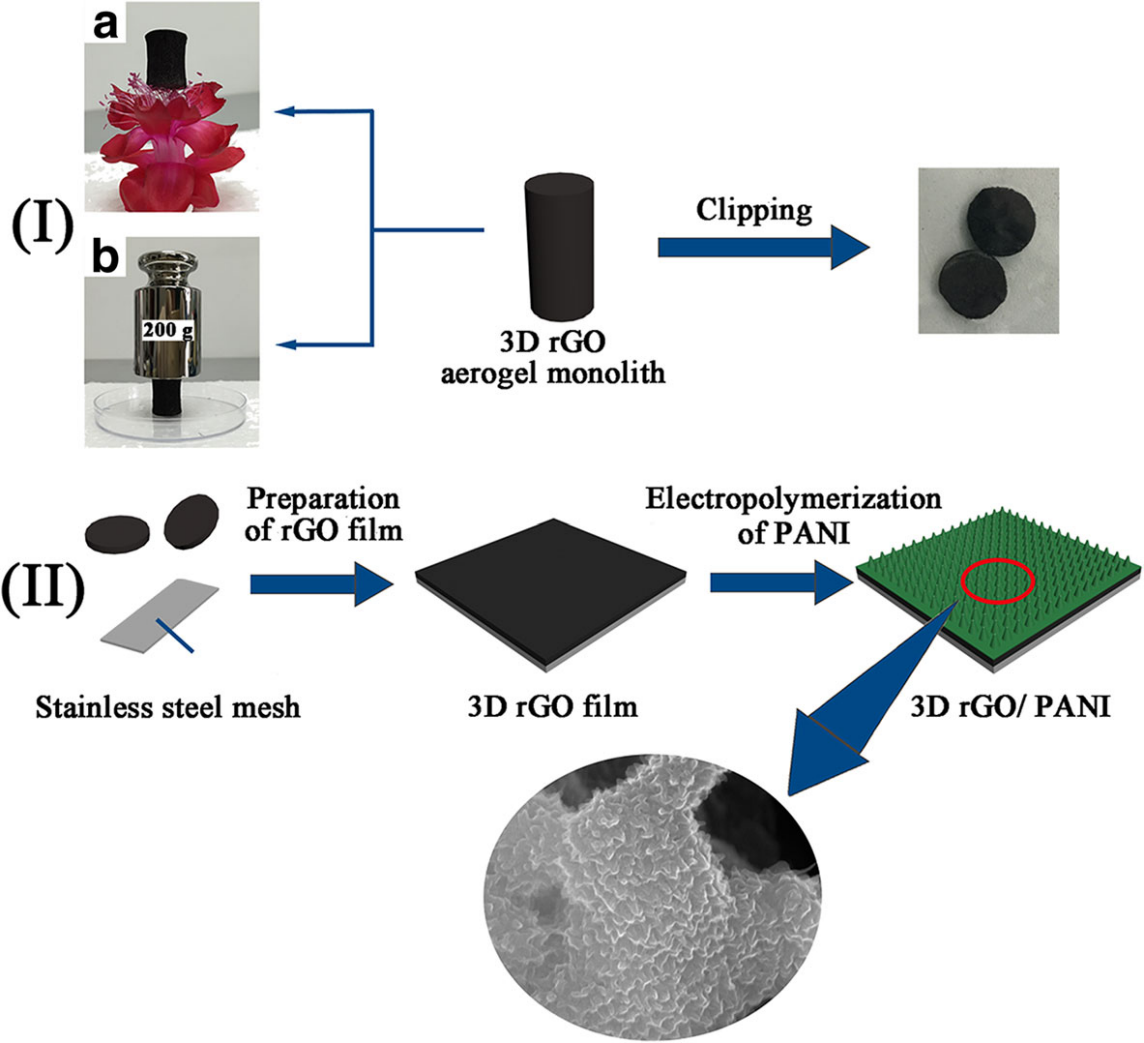
Illustration for fabrication process of (*i*) 3D rGO aerogel slices from the monolith (**a**, **b** - mechanical properties of 3D rGO aerogel) and (*ii*) hybrid composites via a mechanical pressing and electrodeposition method

The fabrication procedure consisted of two-step procedure is illustrated in Fig. 1. Step I: The 3D rGO aerogel monolith (about 47.6 mg) was synthesized via one-step self-assembled hydrothermal process according to the previous reports [16]. In order to test conveniently as the electrode, the 3D rGO aerogel was cut into slices with the thickness about 1 mm. Step II: The as-prepared slices need to be further pressed onto the cleaned stainless steel mesh with the regular square area (1 × 1 cm^2^) by the roller press. With the assistance of the insulating tape, PANI thin film was coated on the surface of 3D rGO aerogel via a galvanostatic electro-polymerization method at a current density of 2 mA cm^-2^. Compared with other techniques to grow PANI nanostructures on the 3D frame, galvanostatic electrodeposition can enable the uniformly growth of PANI arrays on the outside and inside pore surface of the 3D rGO. Moreover, the generating PANI array films can further make the 3D rGO and PANI connected tightly, which is appropriate for the bending property of flexible all-solid-state SCs [18].

**Fig. 2:**
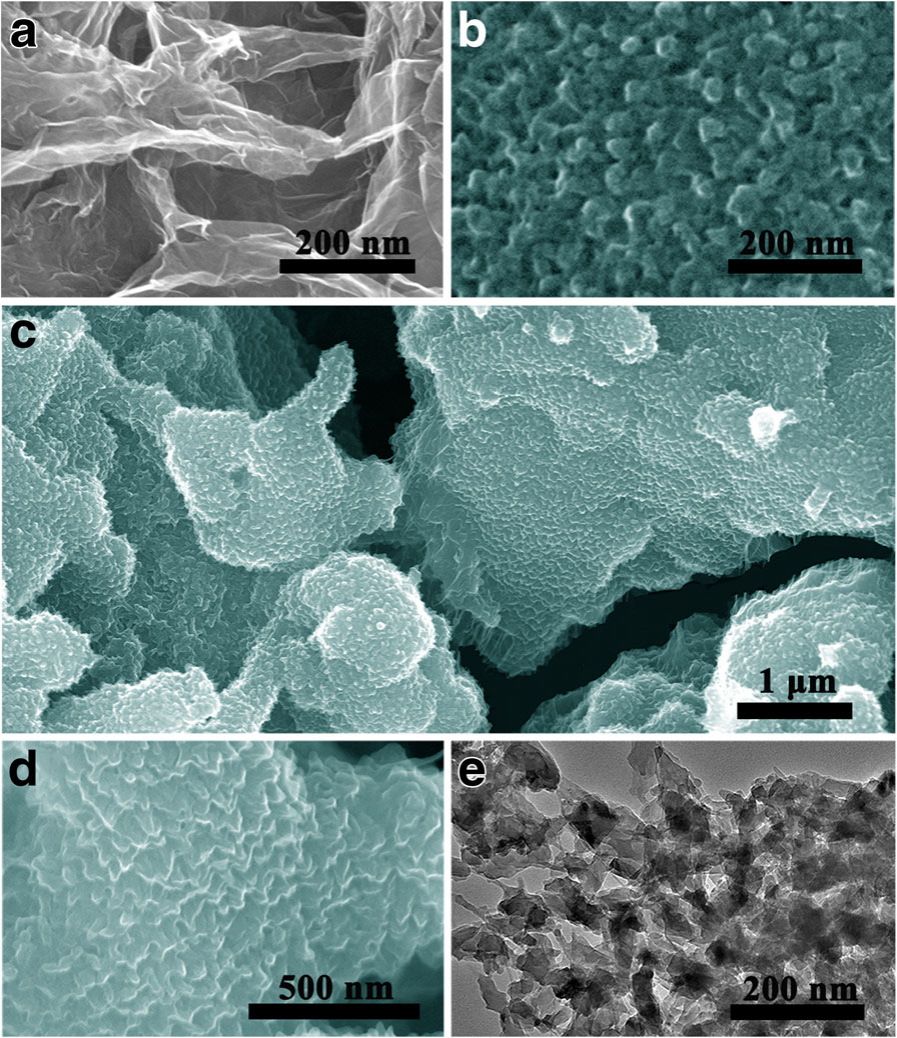
Typical SEM images of (**a**) rGO, (**b**) pure PANI and (**c**, **d**) hybrid composites and at the different magnifications. TEM images of (**e**) hybrid composites

The rGO, pure PANI and hybrid composites were first analyzed by SEM. Figure 2a shows a typical SEM image of freeze-dried rGO, it can be clearly seen that the surface of graphene sheets is relatively smooth, which can serve as suitably substrate for electrodeposited polyaniline arrays with the similar size (Fig. 2b) [19]. From the SEM image of hybrid composites, as displayed in Fig. 2c, d, we can see the PANI nanocones homogeneous and erectly grow on the whole surface of three-dimensional rGO. Through expounding the distributed situation of PANI nanocones, it could be explicitly inferred that the nucleation and growth processes of PANI occurred on the internal surface of the 3D reduced graphene oxide layers. With a closer observation of hybrid composites by TEM, it shows that the PANI nanocones are attached tightly to the reduced graphene oxide layers, which effectively prevent grapheme sheets from aggregating [20]. Interesting, the nanostructure of hybrid composites can be controlled by the electodepositional process. Weak deposition leads to the sparseness and viscousness for PANI film and overdepositing difficulty to realize the synergy effect with rGO, by contrast we find optimal depositing time is 7000 s.

**Fig. 3:**
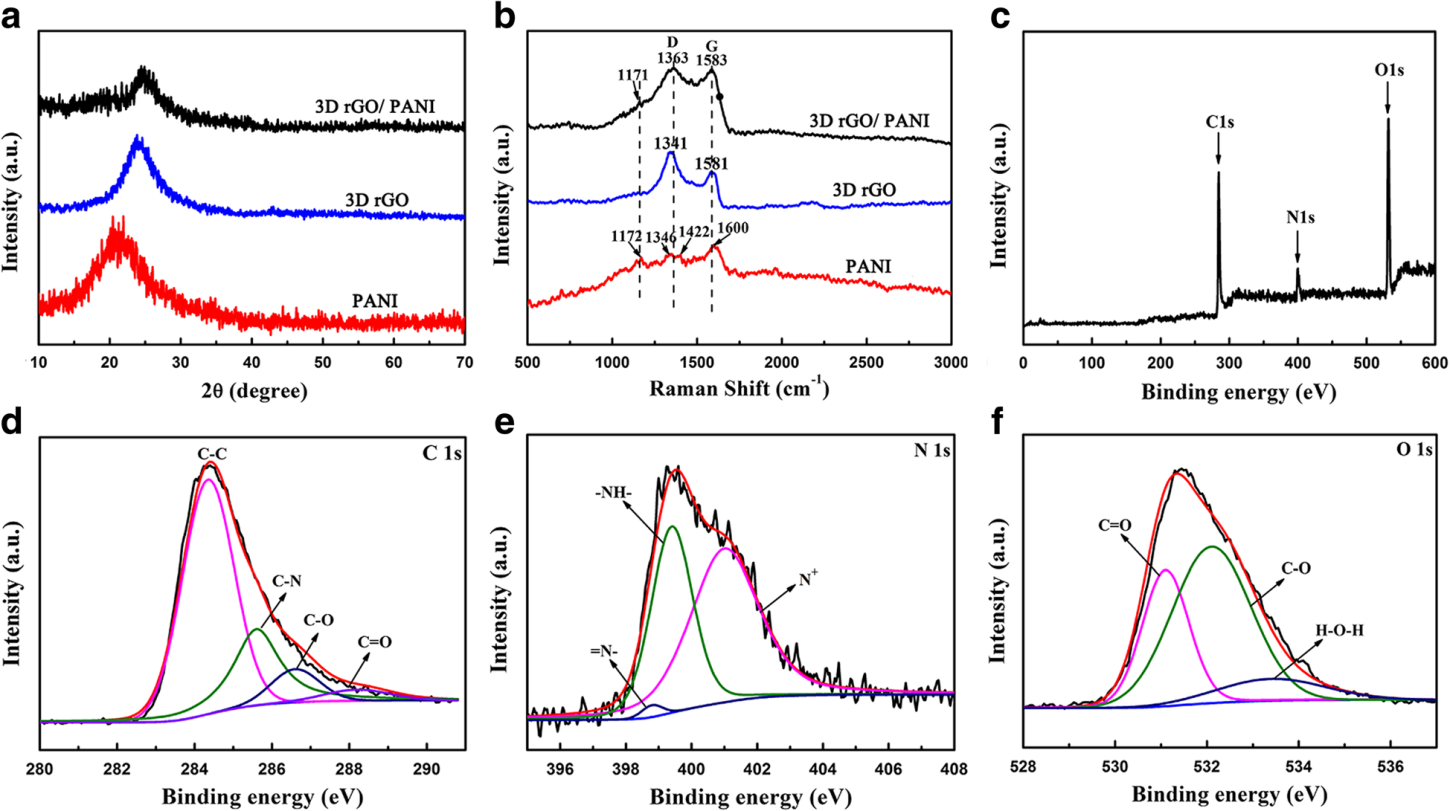
**a** X-ray diffraction (XRD) patterns; **b** Raman spectrum of hybrid composites, PANI and rGO; **c** X-ray photoelectron spectra (XPS) spectrum of hybrid composites composite film; **d**–**f** XPS data of the N 1 s,C 1 s, and O 1 s regions of the hybrid composites, respectively

The crystallization and phase composition of the as prepared materials were also characterized with using XRD as presented in Fig. 3a. For PANI, the diffraction peaks appear at 26°, confirming that the electropolymerized PANI is non-crystal structure with amorphous state [21]. A broad diffraction peak centered at around 21.8° can be observed for rGO, which reveals the presense of graphitic crystal structure [22]. Compared to the electropolymerized PANI and rGO, the hybrid composites composite has a broad peak between 15°-30°, but the most intense peak slightly shifts towards the 26.2°, which can be visually accounted for the superposition of the peaks measured in sample electropolymerized PANI and rGO, respectively. It should be noted that the formed hybrid composites structure is stable enough to use as the electrode material. In order to examine the chemical bonds rather than weak physical adsorption, the obtained samples were further verified by Raman spectral, as shown in Fig. 3b. For rGO, two peaks appers at 1341 cm^-1^ and 1581 cm^-1^ confrom to the D and G bands of the rGO, respectively. The Raman spectrum of the pure PANI presents characteristic peaks at 1172, 1346, 1422^,^ and 1600 cm^-1^ corresponding to the C-H, C-N, C = N and C = C bond [23].For hybrid composites, the D band locates at 1363 cm^-1^ and G band locates at 1583 cm^-1^, respectively [24]. The value of I(D)/I(G) decreases, which indicates the hybrid composites with an ordered structure and the defects of the crystalline structure is fewer than the monomer of PANI and rGO [22].

XPS were used to monitor the surface composition of hybrid composites which was shown in Fig. 3c. Figure 3d exhibits the N1s spectrum, several new types nitrogen-containing functionalities attributed to PANI appeared in the spectrum of hybrid composites. The new group include the quinoid amine groups (=N-), the benzenoid amine nitrogen (–NH–) and positively nitrogen cationic radical (N+) with a binding energy centered at 398.8, 399.4, and 401 eV, respectively [25, 26]. The high ratio of N+ also illustrates that nitrogen protons are successfully doped in hybrid composites and it can improve the electrical conductivity. Simultaneously, a well peak at 285.6 eV can be assigned to the chemical bond C-N in C1s spectrum, found in Fig. 3e, indicates that PANI and 3D rGO are well connected too [27]. Figure 3f provides the O1s spectrum, three peaks at 531.1, 532.1, and 533.4 eV corresponding to the bond of C = O, C-O and H-O-H appeared because of the presence of water or other oxygen molecules groups [28]. All above analysis results prove the PANI were tightly deposited on the surface of 3D rGO, which is beneficial for flexible and tough self-supported structure.

**Fig. 4:**
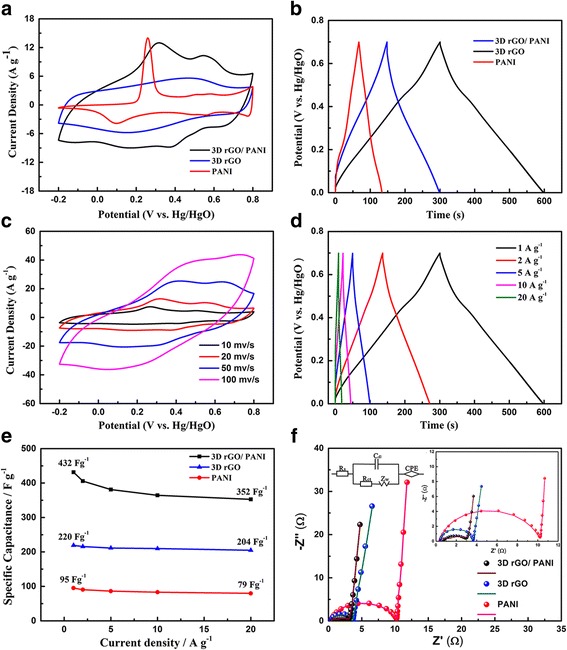
Measurement in three-electrode system. **a** CV curves of hybrid composites, rGO and pure PANI at the scan rate of 20 mV s^-1^ in 1 M H_2_SO_4_. **b** Galvanostatic charge–discharge curves of hybrid composites, rGO and PANI at a current density of 1 A g^-1^. **c** CV curves of hybrid composites composite at different scan rates. **d** Galvanostatic charge–discharge curves of hybrid composites composite at different current densities. **e** Plot of specific capacitance for hybrid composites, rGO and pure PANI electrode at different current densities in 1 M H_2_SO_4_ aqueous electrolyte; **f** Nyquist plots of hybrid composites, rGO and pure PANI electrode in 1 M H_2_SO_4_ aqueous electrolyte. The inset figure shows the magnified high-frequency regions of the Nyquist curves

After elementary characterization of the hybrid composite electrodes, the electrochemical studies were conducted in a three-electrode cell in 1 M H_2_SO_4_ aqueous electrolyte, with a Pt counter electrode and a Hg/Hg_2_SO_4_ reference electrode. The mass loading of the hybrid composites electrodes is about 2.5 mg and the thickness is about 30–40 μm. The CV curves of rGO, pure PANI and hybrid composites were displayed in Fig. 4a. It shows that the closed area of hybrid composites is larger than that of rGO and pure PANI with the same mass. In other words, the capacitive performance of hybrid composites is the best among the three different electrodes. For the CV curves of the rGO, there are two broad peaks at the charge-discharge process, which can be explained that in the rGO exist a small portion of functional groups [29]. These functional groups are favorable for the adhesion of the PANI during electrodeposition process. The CV curve of the pure PANI is a regular shape, revealing the pseudocapacitance behavior of the conducting polymer. Figure 4b shows the GCD curves of the sample at a current density of 1 A g^-1^. For the rGO electrodes, the shape of the charge-discharge curves is isosceles triangle, corresponding to the theoretical model of carbon materials. The specific capacitance (432 F g ^−1^) of the hybrid composites at 1 A g^-1^ is much higher compared to 214 F g ^−1^ of rGO and 98 F g ^−1^ of PANI. To further investigate the electrochemical performance of the hybrid composites, more detailed tests were carried out, as shown in Fig. 4c. The CV curves of hybrid composites composite were implemented at different scan rates [30]. It exhibits that there are several reduction and oxidation peaks in the curves due to the pseudocapacitance by presence of PANI, which is transformed between leucoemeraldine base states and emeraldine salt states of PANI, and emeraldine salt and pernigraniline base states [15]. When the scan rate increases from 1 to 100 mV s^-1^, the cathodic peaks shift positive and the anodic peaks shift negative because of the resistance of the electrode [31]. The GCD curves of hybrid composites at different current densities of 1, 2, 5, and 10 A g^-1^ were provided in Fig. 4d. At the charge-discharge process, an obvious discharge plateau can be observed due to the synergistic effect between double layer capacitance and pseudocapacitance, corresponding to reduced graphene oxide and PANI. Figure 4e illustrates the specific capacitance and rate capability. The specific capacitance of the hybrid composites retains 81.4% when the current density changed from 1 to 20 A g^−1^, demonstrating the hybrid composites with both high specific capacitance and nice rate capability. Then, the electrochemical impedance spectra (EIS) were employed to test the electronic conductivity, as shown in Fig. 4f. The Nyquist plots were consisted of a semicircle part in the high frequency region and an almost straight line part in the low frequency region showed in the insert. The equivalent series resistance (Rs) corresponds to the intercept on the X-axis including intrinsic resistance of ionic resistance of electrolyte, electrode materials, as well as contact resistance between electrode and current collector. The Rs of hybrid composites, rGO and pure PANI is 0.4, 0.45, and 0.33 Ω, respectively, and the interfacial charge transfer resistance (Rct), relates to Faradic reactions and EDLC (Cdl) at the electrode/electrolyte interface, which illustrates the conductivity of the active material [32] and ion behavior of electrolyte ions [33], can be calculated with the value of 1.9, 2.8, and 7.2 Ω, suggesting that as for composites the rGO nanosheets improve the property of ion diffusion and reduce the charge transfer resistance to some extent. The Warburg resistance (Zw) is caused by the frequency dependence of ion diffusion/transport in the electrolyte and the CPE is the constant phase angle element relates to the Zw.

**Fig. 5:**
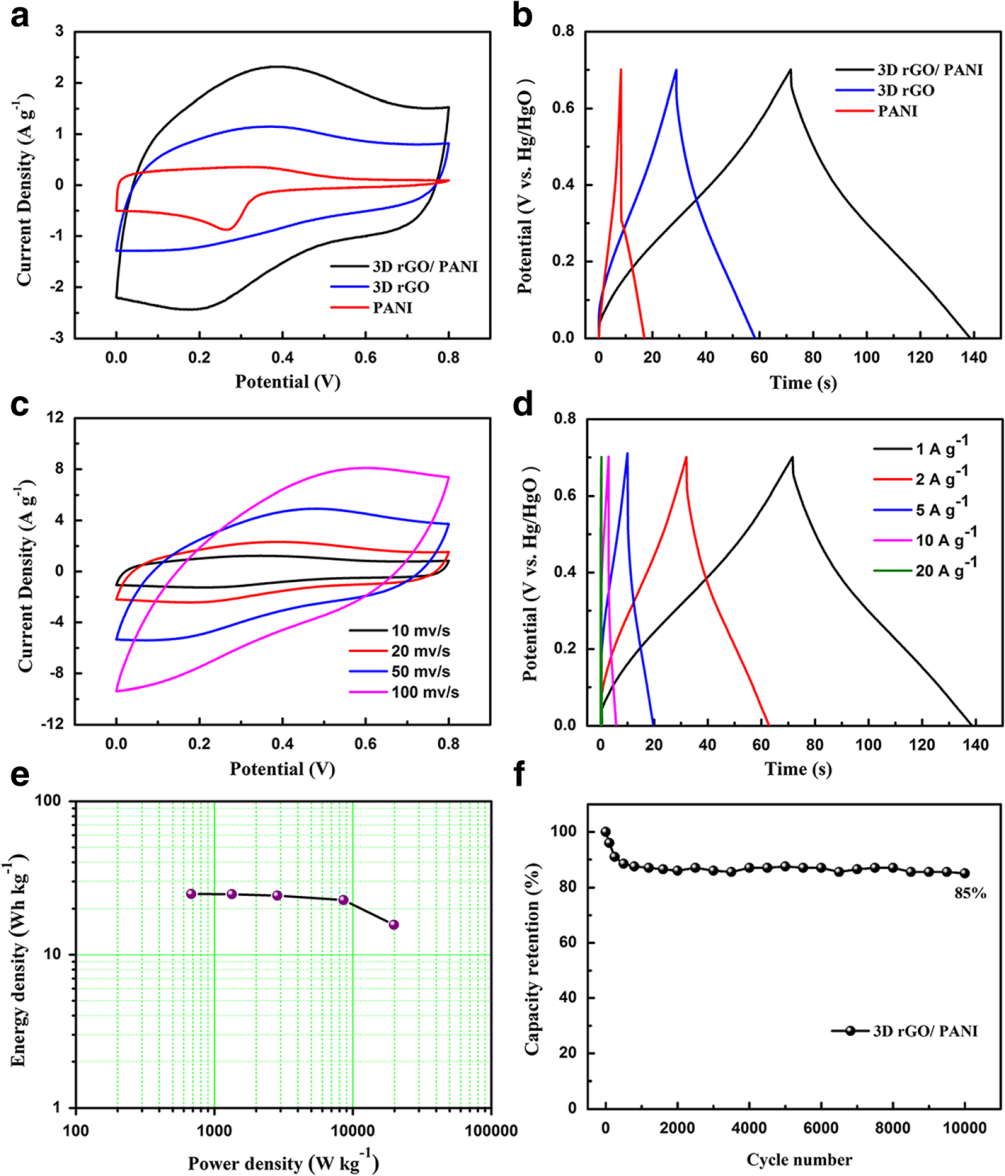
Measurement under the two-electrode system in the form of flexible all-solid-state SCs with PVA-H_2_SO_4_ (**a**) CV curves of hybrid composites, rGO and pure PANI at the scan rate of 20 mV s^-1^. **b** Galvanostatic charge–discharge curves of hybrid composites, rGO and PANI at a current density of 1 A g^-1^. **c** CV curves of hybrid composites composite at different scan rates. **d** Galvanostatic charge–discharge curves of hybrid composites composite at different current densities. **e** Ragone plot of hybrid composites flexible all-solid-state SCs. **f** Cycling stability of hybrid composites flexible all-solid-state supercpacitors at a current density of 1 A g^-1^

Taking advantage of good conductivity of the hybrid composites, we fabricated an all solid-state SCs in PVA-H_2_SO_4_ gel electrolyte. The electrochemical performance of the SCs was tested under the two-electrode system [34]. Figure 5a shows CV curves of the all-solid-state SCs in the range 0 to 0.8 V at the different scan rates. It is clear that the curve area of hybrid composites based SC is larger than that of rGO and pure PANI. Compare with the discharge time of hybrid composites, rGO and PANI based SCs in the GCD curves (Fig. 5b), the hybrid composites possess the longest discharge time, expounding its superior electrochemical performance. Moreover, the smallest IR drops of the hybrid composites based SC indicates it can be used as a promising electrode material for SCs [35]. In order to further investigate the electrochemical performance of the hybrid composites based SC, the CV curves at the various scan rates were tested. In Fig. 5c, the CV curves of the hybrid composites shows the obvious deformation, which can be explained by the inadequate response of electrode materials in the PVA-H_2_SO_4_ gel electrolyte [36]. Figure 5d exhibits GCD curves at the different current densities of 1, 2, 5, 10, and 20 A g^-1^. The Ragone plot of hybrid composites at different scan rates were displayed in Fig. 5e. With increasing power densities, the energy densities reduce by inches. The energy density of the all-solid-state SC based on hybrid composites can reach up to 25 W h kg^-1^ at the power density of 681 W kg^-1^ and remains 15.7 W h^-1^ kg at power density of 20 kW kg^-1^ [37]. The cycle performance is an important parameter for SCs. So Fig. 5f provides the cycle performance of the hybrid composites, taken with 10,000 galvanostatic charge/discharge cycles. Even after 10,000 charge/discharge cycles, 85% of initial value were remained for hybrid composites based SC. This evidences the long cycle life of the SC [38]. There is a sudden decrease of specific capacitance as polymer degradation from the swelling and shrinking during the first 500 cycles, then the synergistic effect between graphene and PANI makes possible hybrid composite film to keep stable in the following cycles. Moreover, 3D conductive network of the 3D rGO film provides the effective strain relaxation of the vertical PANI nanocone arrays during the charge/discharge process. Compared to the composites, pure PANI processed deficient performance in the cycle life commonly. While in the first 2000 cycles, the capacitance retention of PANI decreased rapidly, indicating the inside structure has been collapsed and changed. Moreover, the nanocones array structure of PANI would gradually disappear during charge/discharge process.

**Fig. 6:**
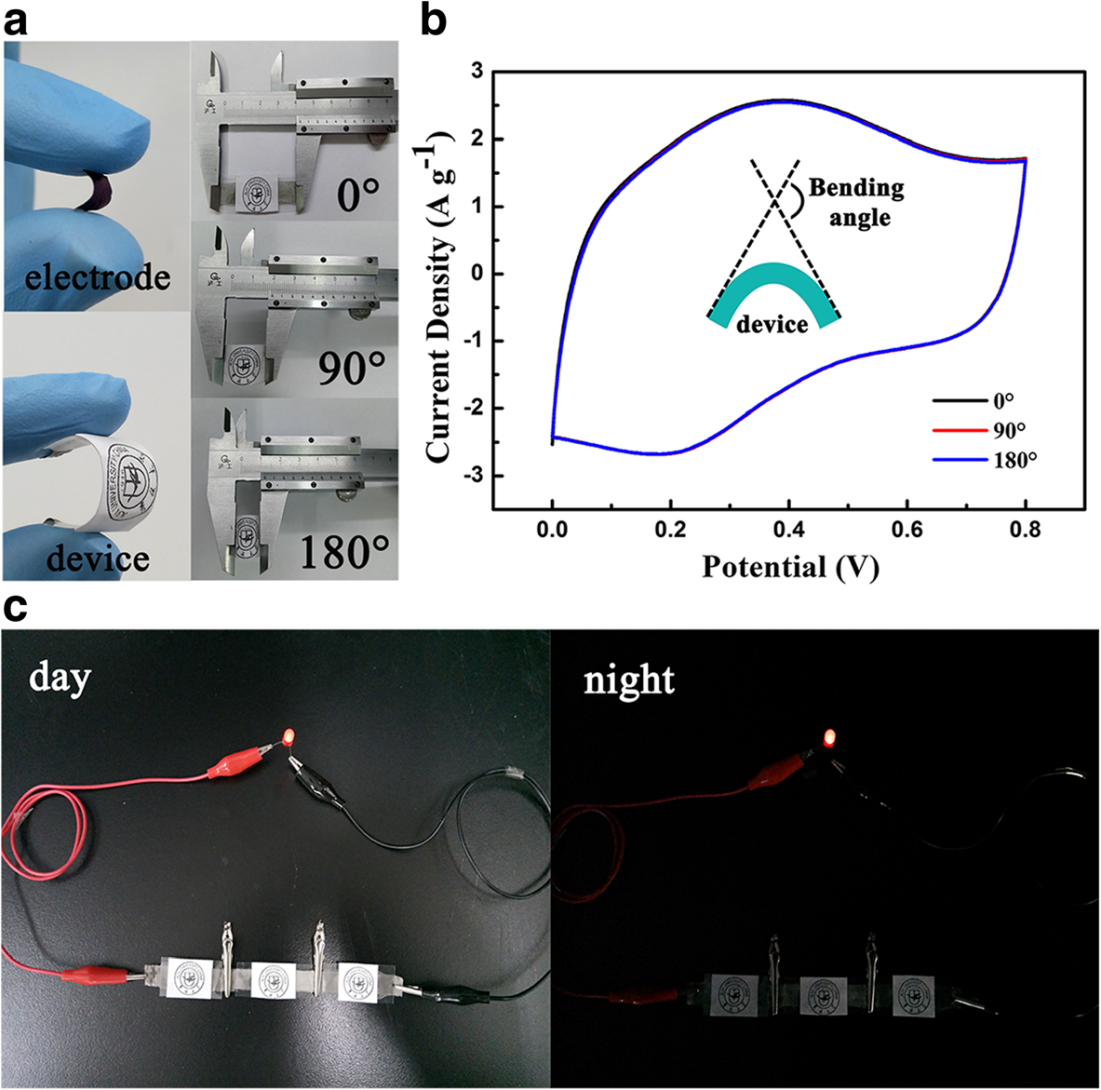
**a** Digital image of hybrid composites electrode, flexible all-solid-state SC and SC in different bending states. **b** CV curves of the hybrid composites based flexible all-solid-state SC at 20 mV/s with different bending angles of 0°, 90°, and 180°. **c** Digital image of red LED which is lighted up by hybrid composites based flexible all-solid-state SC module in day and night condition

In consideration of the practical application of the devices, the flexibility of hybrid composites SCs were also measured. Figure 6a displays the close-ups photos of electrode and flexible all-solid-state SCs (left), while the right part shows the digital photograph of the flexible SC under different bending ratio varying from 0° to 180°. For the bending tests, from the Fig. 6b, we can find the area of the CV curves under various bend conditions exhibit negligible difference, revealing its excellent flexible stability [38, 39]. Moreover, the SCs in series-combinations were integrated to increase the operating voltage. A red LED was lit by the SCs in series under air ambient conditions, suggesting the long-term stability of the hybrid composites based flexible all-solid-state SC, as shown in Fig. 6c [40, 41]. All these flexibility tests and the lighting tests demonstrate it possible for applications in portable electronics [42].

## Conclusions

In conclusion, a flexible all-solid-state SC based on 3D rGO/polyaniline array hybrid composites has been fabricated. The obtained hybrid composites have a specific capacitance of 432 F g^-1^ at the current density of 1A g^-1^, and robust cycling stability with a capacitance retention of 85% after 10,000 charge/discharge cycles. Ulteriorly, the all-solid-state supercapacitor showed a good energy density of 25 W h kg^-1^ and power density of 681 W kg^-1^. The excellent performance of hybrid composites based SCs can be attributed to the special 3D structure and synergistic effect of 3D rGO aerogel and PANI arrays. In addition, the fabricated SCs have superior flexibility and outstanding stability under different bending states. Considering the combined high mechanical and electrochemical properties, the hybrid composite-based flexible all-solid-state SC are particularly promising for the wearable electronics.
